# Geospatial Analysis of Opioid Dispensing Patterns in California: A 2021 Real-World Study

**DOI:** 10.3390/healthcare11121732

**Published:** 2023-06-13

**Authors:** Hongxia Lu, Jianwei Zheng, Yun Wang

**Affiliations:** 1 Department of Pharmaceutical Economics and Policy, School of Pharmacy, Chapman University, Irvine, CA 92618, USA; hongxia_lu@med.unc.edu (H.L.); jzheng@chapman.edu (J.Z.); 2 School of Medicine, University of North Carolina at Chapel Hill, Chapel Hill, NC 27599, USA

**Keywords:** geospatial analysis, opioid dispensing, opioid misuse and abuse, neighborhood characteristic

## Abstract

The misuse and abuse of opioids has become a serious public health threat in the United States. The state of California has been hit particularly hard by the opioid epidemic, with a noticeable increase in opioid-related fatalities and hospitalizations. This brief report paper aims to contribute to the growing literature by conducting a geospatial analysis of opioid dispensing patterns in California in 2021. The primary objective was to identify areas characterized by high-risk opioid dispending patterns and explore possible contributing factors. This retrospective study analyzed data from over 7 million records of opioid and benzodiazepine prescriptions dispensed by outpatient pharmacies in California in 2021. A series of generalized linear regression models was employed to assess the impact of neighborhood characteristics on opioid recipients and high-risk opioid dispensing. The study defined high-risk opioid dispensing behavior as: (1) multiple provider episodes, (2) overlapping opioid prescriptions for seven or more days, (3) overlapping opioid and benzodiazepine prescriptions for seven or more days, and (4) a high standardized dosage of opioid prescriptions per month. The study identified variables associated with high-risk opioid dispensing behaviors, including age, population density, income, and housing-related variables, as well as marital status and family-related variables. The study uncovered that there are noticeable disparities in opioid dispensing among different racial and ethnic groups within California. The findings indicated a correlation of high-risk dispensing indicators with certain demographic and socioeconomic factors. There was a substantial regional variation in opioid dispensing practices, with certain rural areas having higher rates of opioid prescriptions than urban areas.

## 1. Introduction

The opioid epidemic has been a major public health crisis in the United States, and California has not been immune to its devastating effects [1]. Friedman et al. [2] studied the relationship between race/ethnicity and income patterns and opioid prescription rates from 2011 to 2015 in California. Current national trends reveal that, since 2010, heroin and illicitly manufactured fentanyl [3,4,5] have led to significant increases in deaths attributed to opioid overdose rather than opioids directly. Opioid prescriptions declined dramatically from 2016 to 2019, with there being a 44% decline in opioid prescriptions to treat moderate to severe pain [6]. The landscape of opioid dispensing has undergone significant changes due to the COVID-19 pandemic. Poor urban neighborhoods and Black and Hispanic communities were hit hard, while affluent suburban White communities also experienced a rise in overdose deaths [7]. Efforts are needed to address high-risk opioid dispensing behaviors and understand the neighborhood characteristics associated with these behaviors to inform interventions at local, regional, and national levels, particularly within the novel context of the COVID-19 pandemic. These insights can assist researchers to better understand the opioid crisis during the pandemic period and identify the unique challenges faced by individuals and communities during this time.

Prescription Drug Monitoring Programs (PDMPs) are state electronic databases that track individual-level controlled substance prescriptions using pseudo-identifiers to differentiate records without revealing an individual’s identity. When providers dispense controlled substances to patients, they must enter the prescription into the state PDMP and query the PDMP database to check if patients are “doctor shopping” controlled substance prescriptions [1]. Data routinely collected by PDMPs can be used to identify high-risk opioid use in adults. Existing metrics [8,9,10] of high-risk opioid dispensing include a large volume of opioid prescriptions; long durations of opioid prescription; high-dosage and extended-release/long-acting formulation prescriptions; opioid prescriptions co-prescribed with codeine, tramadol, gabapentin, pregabalin, and benzodiazepine; and receiving opioids from multiple prescribers/pharmacies.

Prior studies have demonstrated significant geographic variation in opioid overdoses, with certain areas experiencing higher rates than others [11,12]. According to the California Overdose Surveillance Dashboard managed by the California Department of Public Health (CDPH) and the California Department of Health Care Access and Information (HCAI), the Mendocino, Trinity, and Alpine counties reported higher age-adjusted rates of opioid overdose deaths than other counties in California in 2021 [13]. The US Center for Disease Control and Prevention (CDC) reported that the Sutter, Tuolumne, Butte, and Shasta counties had higher dispensing rates than other counties in California in 2020 [14]. Individual characteristics such as age and sex have also been identified to be associated with high-risk opioid dispensing [15].

Understanding the characteristics of high-risk neighborhoods is important in prioritizing limited prevention and intervention resources [16]. Previous studies identified a range of dimensions of socioeconomic predictors for opioid overdose deaths in neighborhoods, including education, income and wealth, residential stability, race/ethnicity, social isolation, and occupational status [17]. The highest rate of opioid-related deaths occurred in neighborhoods with high economic hardship (36.9 per 100,000 population) compared to medium-(20.5) and low-(12.3) hardship neighborhoods. However, these patterns were not consistent across racial/Hispanic ethnicity subgroups [18]. Adolescents in socially disorganized neighborhoods and also those in neighborhoods with lower levels of social capital were more likely to report prescription drug misuse [19]. However, little was known about the neighborhood characteristics of opioid dispensing in adults.

To better understand the distribution and utilization of opioid medications in California, this work presents a geospatial analysis of opioid dispensing patterns using real-world data from 2021. This study builds on the existing literature by using geospatial analysis to examine opioid dispensing patterns in California and using a series of generalized linear regression models to identify the demographic and socioeconomic factors that may influence high-risk dispensing patterns.

## 2. Methods

### 2.1. Data

The opioid prescription data were derived from California’s Controlled Substance Utilization Review and Evaluation System (CURES)—prescription drug monitoring programs (PDMP), which is operated by The California Department of Justice (CADOJ) [20]. CURES is a state-operated database that collects information on Schedule II-V prescription drugs dispensed by outpatient pharmacies in California. Opioids and benzodiazepines are Schedule II-IV drugs and therefore are included in the CURES database. Data provided by the California Department of Justice and used in this study were completely de-identified to comply with the Health Insurance Portability and Accountability Act (HIPAA) Privacy Rule. All direct personal identifiers were removed, including but not limited to names, addresses, and social security numbers. In addition to this, a stringent data use agreement was in place to prevent attempts to identify individual patients. To ensure the privacy and security of the sensitive data, all personally identifiable information was anonymized, and the data was stored in a secure, password-protected environment. The data included information about the year of birth and the sex of the patient, product name regarding the prescription, National Drug Code (NDC), form and strength of the drug, number of metric units dispensed, estimated number of the days the medication will cover, date the prescription was filled, number of the fill of the drug, number of authorized refills, and code identifying the type of payment (private pay, Medicaid, Medicare, commercial insurance, and others). To prepare the data for analysis, we performed an initial cleaning process, which involved removing any records with missing or inconsistent values.

The study sample consisted of California residents who were 18 to 100 years old with at least one record of oral opioid prescription in 2021. We intended to only investigate adult individuals since adolescents may present different patterns in terms of opioid misuse and abuse. We identified the morphine milligram equivalents (MME) conversion factor from the Centers for Disease Control and Prevention (CDC) file of National Drug Codes for Opioid analgesics, and Linked Oral Morphine Milligram Equivalent Conversion Factors, 2020 Version [21]. In line with the existing literature, we converted different potency of opioids to MME by multiplying the strength by the quantity of the prescription and then adjusting this dose using MME conversion factors [22,23]. We exclude non-California residents to examine the effects of the pandemic on only California-based opioid users. We excluded those who were aged above 100 years old [24]. We limited our study sample to oral tablets, capsules, and lozenges/troches. Hence, formulations including or consisting of powder, elixir, solution, suppository, extended-release patch, liquid, spray, syrup, and tincture [25] were excluded from the study. In addition, we excluded dispensing records of transactions with erroneous or extreme values (MME ≥ 360 mg per transaction) [23].

The US Zip Codes Database (Pareto Software^TM^, version 2023) is a comprehensive database that provides detailed information on zip codes in the United States. This database contains over 42,000 zip codes, covering all 50 states, and includes information on geographical coordinates, population, area code, time zone, and more. With this database, users can quickly and easily search for zip codes based on a range of criteria, such as population density, income, or age.

### 2.2. Measures

We examined four indices of high-risk opioid dispensing: (1) multiple provider episodes [10], (2) overlapping opioid prescriptions for ≥7 days [26,27,28], (3) overlapping opioid and benzodiazepine prescriptions for ≥7 days [29,30], and (4) a high standardized dosage of opioid prescriptions [31,32]. These indices were selected because they either strongly suggested doctor shopping behavior, such as opioid users seeing multiple providers, including prescribers and pharmacies within a single episode [33,34], or were associated with opioid abuse or misuse, such as physicians dispensing opioids or benzodiazepine without paying appropriate attention.

The total standardized dosage of opioids was calculated as the potency of opioids by MME per dispensing. Multiple provider episodes were defined as opioid users receiving opioids from two or more prescribers and pharmacies within a 30-day interval [10]. Overlapping opioid prescriptions for ≥7 days were defined as a binary variable with two opioid prescriptions that overlapped by ≥7 days [26,27,28]. Overlapping opioid and benzodiazepine prescriptions for seven or more days were defined as a binary variable with one opioid prescription and one benzodiazepine by ≥7 days [29,30]. The high standardized dosage of opioid prescriptions measurement was defined as a binary variable with a daily dose exceeding 120 MMEs [31,32].

For the total standardized dosage of opioids, all opioid users’ total standardized dosage (MMEs) was summed together per month. For the four remaining measures, the monthly count of opioid users who presented high-risk behavior was used.

### 2.3. Method

We linked two datasets that included information on opioid prescriptions and the demographic characteristics of patients across multiple geographical regions. We first conducted exploratory data analysis to examine the distribution and patterns of the variables. We then fit a series of generalized linear regression (GLR) models to analyze the relationship between high-risk opioid dispensing behaviors and demographic characteristics while controlling for potential confounders such as demographic characteristics and health status. For variables that sum up to 100, such as the race, education, and marital status variables, one category from each of those variables was removed to avoid co-linearity.

The GLR models allowed us to model the continuous outcome of high-risk opioid dispensing behaviors as a function of the predictor variables while accounting for the non-normality of the outcome variable. We assessed the goodness of fit of the models using appropriate diagnostic tests, such as residual analysis, and model selection criteria, such as the Akaike Information Criterion (AIC).

### 2.4. Statistical Analyses

Opioid recipients’ characteristics were described using percentages, means with SDs, or medians, as appropriate. One-sample test for proportions, two-sample *t*-test, two-sample test for proportions, and Fisher’s exact test were adopted to test the difference in the sample numbers, average ages, and genders. To analyze the association between high-risk opioid dispensing behaviors and neighborhood characteristics, we implemented generalized regression models to estimate the effect size of associations. We investigated a total of 41 candidate neighborhood characteristics variables and used a backward stepwise method to identify the significant variables based on the AIC (Akaike Information Criterion) value. Insignificant variables were excluded from the final model. A *p*-value less than 0.05 is generally considered statistically significant. However, some variables in the final models may not have a *p*-value of less than 0.05. This is because the variable selection was performed in a backward stepwise fashion. If removing a variable resulted in an increase in AIC value, the variable was kept in the final model, even if its *p*-value was greater than 0.05. A detailed description of the model specification is presented in the Supplementary Materials.

All analyses were conducted using Stata/SE 17 (StataCorp LP, College Station, TX, USA), R 3.5, and Python 3.8.

## 3. Results

This study analyzed data from California residents aged 18 to 100 with at least one opioid prescription record in the state’s PDMP in 2021. The PDMP data included 7,776,640 records of oral opioids or benzodiazepines drugs dispensed by outpatient pharmacies. The study included 1,300,171 opioid recipients (shown in Table 1), with a median age of 64 for both males and females. The mean age for males was 62.4, and the mean age for females was 63.29, with a standard deviation of 14.12 and 15.06, respectively. Out of all recipients, 208,454 (8.87%) were exposed to opioid drugs for over 180 days. In our observation, 7.2% of recipients experienced multiple provider episodes, 22.33% had overlapping opioid prescriptions for ≥7 days, 8% had overlapping opioid and benzodiazepine prescriptions for ≥7 days, and 1.56% had a high standardized dosage of opioid prescriptions.

**Figure 1:**
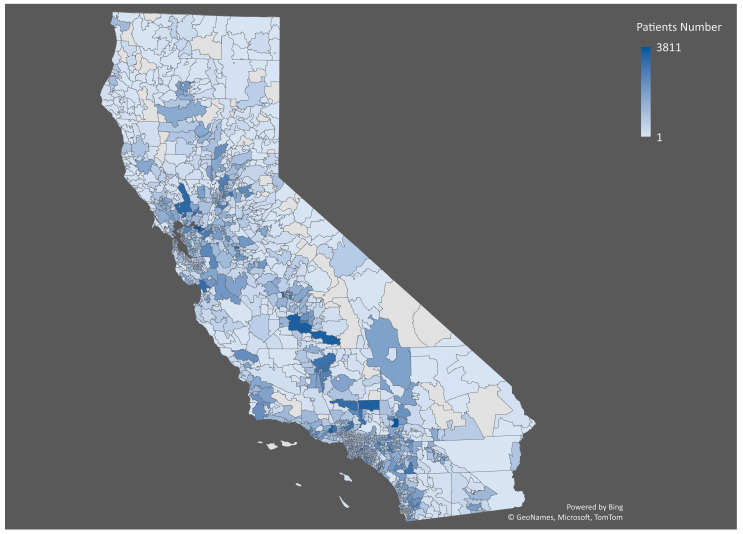
Geographic distribution of opioid recipients by zip code in California in 2021 (the map shows the number of opioid recipients by zip code, with darker colors indicating a higher number of recipients).

**Figure 2:**
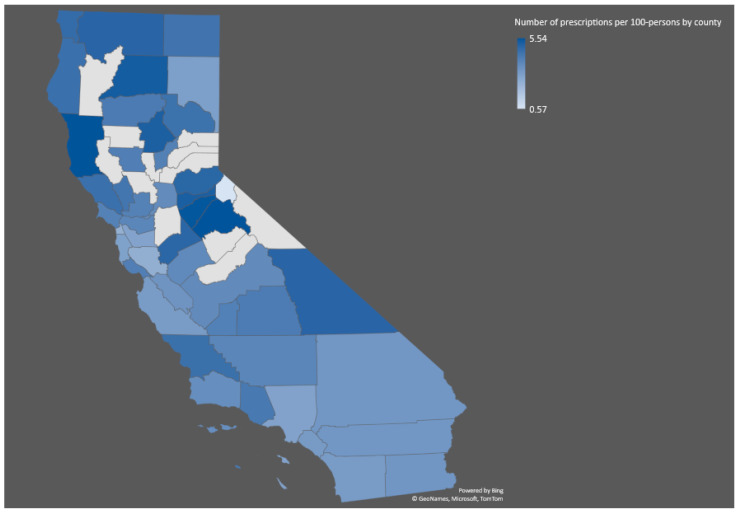
Geographic distribution of opioid prescriptions by county in California in 2021 (the map shows the number of dispensed opioid prescriptions per 100 persons by county).

The distribution of opioid recipients in California varies widely by region (Shown in Figure 1 and Figure 2). Some of the areas with the highest rates of opioid prescriptions include rural areas in the Central Valley and Northern California (Figure 2). In contrast, some urban areas, such as San Francisco and Los Angeles, have lower rates of opioid prescriptions. The highest number of patients is in zip code 94565—the city of Pittsburg—with 3811 recipients, followed by zip code 92345 (Hesperia) with 3742 recipients, zip code 93065 (Simi Valley) with 3587 recipients, zip code 93274 (Tulare) with 3574 recipients, and zip code 93257 (Porterville) with 3553 recipients (Figure 1).

The associations of the 41 candidate neighborhood characteristics with high-risk opioid prescribing outcomes that were used for statistical analysis are presented in Table 2. Of the candidate variables investigated, 21 were significantly associated with incidents of a high standardized dosage of opioid prescriptions, 23 with the occurrence of overlapping opioid prescriptions for ≥7 days, 24 with incidents of overlapping opioid and benzodiazepine prescriptions for ≥7 days, and 24 with multiple provider episodes. The percentage of farmer population was found to have an inverse relationship with all four measures, suggesting that these high-risk opioid dispensing behaviors are less prevalent in rural areas. Other common significant variables that were negatively associated with the four types of opioid misuse and abuse behaviors are the percentage of residents without health insurance, the median age of the residents, the percentage of “married” and “never married” residents, and the percentage of residents who are of Asian descent. This suggests that residents who have health insurance, whose marital status is either “married” or “never married”, and those who are of Asian descent are less likely to exhibit opioid misuse and abuse behaviors. The number of housing units (or households) is the only significant variable that is positively associated with all four measures, which suggests that the more housing units in the zip code area, the more incidents of high-risk opioid seeking and dispensing. The detailed descriptions of the 41 variables are listed in Table S1 in the Supplementary Materials.

## 4. Discussion

The present study provides valuable insights into high-risk dispensing behaviors associated with opioid use in California. By analyzing PDMP data, the study identified several high-risk dispensing indicators, including multiple provider episodes, overlapping opioid prescriptions for ≥7 days, overlapping opioid and benzodiazepine prescriptions for ≥7 days, and a high standardized dosage of opioid prescriptions.

It is important to distinguish between individuals who have been prescribed substantial amounts of opioids and those who actually misuse them. In the literature, older adults are more likely to experience chronic pain; thus, they are prescribed more opioids [35]. However, in our study, older communities report a low rate of high-risk opioid dispensing behaviors. Likewise, those in population-dense neighborhoods are unlikely to experience multiple provider episodes, overlapping opioid prescriptions, and the concurrent use of opioids and benzodiazepines. Communities under the zip codes with a higher prevalence of graduate degrees are associated with lower risks of overlapping opioid prescriptions, multiple provider episodes, the concurrent use of opioids and benzodiazepines, and high-dosage opioids. However, they may be more likely to experience multiple provider episodes. Rural areas with more farmers tend to experience lower incidences of high-risk dispensing behaviors. The results of our study echo that of a study from 2017, in which it was stated that higher rates of overdose-related deaths are experienced in urban areas, while higher rates of dispensing occur in rural areas [36]. Asian and Hispanic communities are protective factors in terms of high-risk opioid dispensing behaviors, while the White community, which has traditionally been associated with higher rates of opioid prescriptions [2], did not show a tendency toward high-risk opioid dispensing, except for overlapping opioid prescriptions. Interestingly, neighborhoods with a higher percentage of Pacific Islanders have a higher likelihood of high-risk opioid dispensing. However, the present study has certain limitations. First of all, as a retrospective study, it is vulnerable to potential biases and confounding variables. Secondly, certain vital variables were not available in the databases and therefore were not incorporated in this study. For example, illicit opioid consumption is a significant factor contributing to the opioid epidemic, and high-risk dispensing practices may also be influenced by comorbidities, mental health ailments, and social determinants of health. Lastly, since our study cohort excluded residents who are under the age of 18, bias or inconsistency may have been introduced in the study in the terms of education and income levels.

## 5. Conclusions

This study offers valuable insights into high-risk opioid dispensing behaviors in California during the pandemic, shedding light on significant variables that influence these behaviors. Our findings suggest that addressing factors such as health insurance coverage, marital status, and cultural backgrounds may contribute to reducing opioid misuse and abuse behaviors. Additionally, this study highlights the importance of understanding the relationship between housing unit density and opioid-related issues, indicating that areas with a larger number of housing units may require targeted interventions to mitigate the risk of high-risk opioid behaviors. Although our findings have implications for targeted interventions and the development of policies aimed at reducing high-risk dispensing behaviors and preventing opioid misuse and overdose, it is important to note that further research is required to better comprehend the broader context of opioid use and devise comprehensive strategies to effectively address the ongoing opioid epidemic.

## Figures and Tables

**Table 1:** Summary Statistics of Demographic Data and Baseline Characteristics of All Study Subjects.

**Baseline Characteristics and Dispensing Outcomes (Year 2021)**	
Number of opioid recipients	1,300,171
Number of dispensing recorders	7,776,640
Number of prescribers	98,408
Male	548,446 (42.18%)
Male Age (median, mean, std)	64, 62.40, 14.12
Female	751,725 (57.82%)
Female Age (median, mean, std)	64, 63.29, 15.06
The number of recipients who were exposed to opioid drugs over 180 days (*n*, %)	208,454 (8.87%)
**The Number of Recipients Who Have a High-Risk Dispensing Indicator**	
(1) Multiple provider episodes (*n*, %)	93,462 (7.2%)
(2) Overlapping opioid prescription for ≥7 days (*n*, %)	290,351 (22.33%)
(3) Overlapping opioid and benzodiazepine for ≥7 days (*n*, %)	104,357 (8%)
(4) High standardized dosage of opioid prescriptions (*n*, %)	20,236 (1.56%)

**Table 2:** Associations of neighborhood characteristics with high-risk opioid dispensing outcomes.

Variable	Multiple Provider Episodes (95% CI)	Overlapping Opioid Prescription for ≥7 Days (95% CI)	Overlapping Opioid and Benzodiazepine for ≥7 Days (95% CI)	High Standardized Dosage of Opioid Prescriptions (95% CI)
Median Age	−5.5622 (−8.544, −2.5803)	−6.72 (−8.7449, −4.6951)	−7.7471 (−10.8811, −4.613)	−1.7757 (−2.6644, −0.887)
% Charitable Givers	3.0683 (0.6167, 5.5200)	4.0389 (2.3577, 5.72)	10.7542 (8.3241, 13.1843)	Not Significant
Median Commute Time	Not Significant	Not Significant	−2.229 (−4.2921, −0.1659)	−0.422 (−0.9977, 0.1536)
Density	−0.0273 (−0.0348, −0.0198)	−0.0132 (−0.0182, −0.0082)	−0.0191 (−0.0267, −0.0115)	Not Significant
% Disabled	Not Significant	2.0875 (−0.7391, 4.914)	5.2299 (0.7059, 9.7539)	1.2025 (−0.0422, 2.4472)
% Divorced	Not Significant	Not Significant	Not Significant	Not Significant
% Education Bachelors	Not Significant	−6.076 (−7.9764, −4.1755)	−9.884 (−12.8724, −6.8957)	−2.682 (−3.5967, −1.7672)
% Education College or Above	Not Significant	Not Significant	Not Significant	Not Significant
% Education Graduate	3.2643 (−0.3551, 6.8837)	−4.2127 (−5.9054, −2.52)	−8.2961 (−10.864, −5.7282)	−1.9957 (−2.813, −1.1785)
% Education Highschool	10.713 (7.6595, 13.7665)	Not Significant	Not Significant	−0.9965 (−1.8982, −0.0947)
% Education Less Highschool	12.0349 (8.8476, 15.2221)	−1.4927 (−3.1816, 0.1962)	−3.906 (−6.3666, −1.4454)	Not Significant
% Education Some College	10.7529 (7.5149, 13.9909)	Not Significant	Not Significant	Not Significant
% Family Dual Income	Not Significant	Not Significant	Not Significant	Not Significant
Average Family Size	Not Significant	Not Significant	Not Significant	Not Significant
% Farmer	−11.2116 (−18.1447, −4.2785)	−8.2954 (−12.9195, −3.6713)	−12.6309 (−19.9376, −5.3241)	−2.73 (−4.8246, −0.6355)
% Health Uninsured	−9.3876 (−13.6014, −5.1737)	−4.3291 (−7.1293, −1.5289)	−5.7225 (−10.238, −1.2069)	−2.3292 (−3.6186, −1.0398)
% Hispanic	−2.8474 (−4.1803, −1.5144)	−0.8857 (−1.7411, −0.0304)	Not Significant	−0.6315 (−0.974, −0.289)
% Home Ownership	1.3882 (−0.054, 2.8304)	0.6639 (−0.2548, 1.5827)	Not Significant	0.468 (0.0996, 0.8364)
Median Home Value	−0.0001 (−0.0001, 0.0000)	0.0000 (−0.0001, 0.0000)	Not Significant	Not Significant
Housing Units	0.0574 (0.0553, 0.0594)	0.0356 (0.0305, 0.0407)	0.0647 (0.057, 0.0723)	0.0145 (0.0121, 0.0168)
% Income Household $150 K Over	3.7928 (0.4511, 7.1344)	2.0254 (0.2468, 3.804)	Not Significant	1.7441 (1.0319, 2.4563)
Median Household Income	0.0016 (0.0001, 0.0031)	Not Significant	Not Significant	Not Significant
% Household Income Under $5 K	Not Significant	Not Significant	Not Significant	Not Significant
Median Individual Income	−0.0018 (−0.0039, 0.0003)	−0.0011 (−0.0024, 0.0001)	−0.0029 (−0.0047, −0.0011)	−0.0004 (−0.001, 0.0001)
% Labor Force Participation	Not Significant	−1.2225 (−2.7464, 0.3013)	−2.6415 (−5.0544, −0.2287)	Not Significant
% Limited English	Not Significant	Not Significant	Not Significant	−0.592 (−1.2068, 0.0228)
% Male	−7.593 (−11.4132, −3.7728)	Not Significant	−3.1298 (−7.2258, 0.9661)	Not Significant
% Married	−8.7214 (−12.2559, −5.187)	−4.7524 (−7.1951, −2.3097)	−5.3964 (−9.2797, −1.5131)	−1.3759 (−2.4982, −0.2535)
% Never Married	−8.1634 (−12.1485, −4.1783)	−5.7531 (−8.3803, −3.1259)	−6.6714 (−11.0447, −2.298)	−1.6997 (−2.8934, −0.5059)
Population	Not Significant	−0.0015 (−0.0031, 0.0002)	−0.0061 (−0.0085, −0.0037)	−0.0015 (−0.0022, −0.0007)
% Race Asian	−5.5453 (−6.6683, −4.4223)	−1.8246 (−2.9029, −0.7463)	−4.3852 (−5.5416, −3.2288)	−1.2717 (−1.6369, −0.9066)
% Race Black	Not Significant	Not Significant	−5.3587 (−7.3606, −3.3569)	Not Significant
% Race Native	−4.2608 (−8.084, −0.4376)	Not Significant	−4.8884 (−8.8992, −0.8776)	−0.994 (−2.1427, 0.1546)
% Race Other	−1.5957 (−3.2122, 0.0209)	Not Significant	−2.9232 (−4.4787, −1.3676)	Not Significant
% Race Pacific	54.9469 (31.8808, 78.013)	15.6273 (0.086, 31.1686)	Not Significant	7.1838 (0.1787, 14.1889)
% Race White	Not Significant	1.4252 (0.6343, 2.2162)	Not Significant	Not Significant
Median Rent Burden	Not Significant	−0.8263 (−1.8684, 0.2158)	−2.4403 (−4.1252, −0.7553)	Not Significant
Median Rent	Not Significant	Not Significant	Not Significant	Not Significant
% Self Employed	Not Significant	Not Significant	4.5369 (1.1474, 7.9264)	0.8391 (−0.1078, 1.786)
Unemployment Rate	−3.8167 (−7.5801, −0.0533)	Not Significant	Not Significant	Not Significant
% Veteran	Not Significant	Not Significant	−11.4975 (−17.6392, −5.3558)	Not Significant

Note: A positive coefficient implies a positive association, while a negative coefficient indicates negative association.

## Data Availability

The opioid prescription data were derived from California’s Controlled Substance Utilization Review and Evaluation System (CURES)-prescription drug monitoring programs (PDMP), and the Zip Code level data were extracted from https://simplemaps.com/data/us-zips (accessed on 15 April 2023).

## Supplementary Materials

The following supporting information can be downloaded at: https://www.mdpi.com/article/10.3390/healthcare11121732/s1, Table S1: Variables and their descriptions; Figure S1: QQ-Plot of Overlapping Opioid Prescription for ≥7 Days; Figure S2: QQ-Plot of Overlapping Opioid and Benzodiazepine for ≥7 days; Figure S3: QQ-Plot of Multiple Provider Episodes; Figure S4: QQ-Plot of High standardized Dosage of Opioid Prescriptions.
