# Supplementary material for: Geospatial Analysis of Opioid Dispensing Patterns in California: A 2021 Real-World Study
Source: Healthcare (Basel). 2023 Jun 13;11(12):1732. doi: 10.3390/healthcare11121732 (PMC10298226; doi:10.3390/healthcare11121732)

Supplementary Materials

Geospatial Analysis of Opioid Dispensing Patterns in California: A 2021 Real World Study

1. Variables

Table S1: Variables and their descriptions

Variable	Description
Median Age	The median age of residents in the zip code.
% Charitable Givers	The percentage of households reporting charitable giving on their 2016 tax return.
Median Commute Time	The median commute time of resident workers in minutes.
Density	The estimated population per square kilometer.
% Disabled	The percentage of residents who report a disability.
% Divorced	The percentage of residents divorced.
% Education Bachelors	The percentage of residents with a bachelor's degree (or equivalent) but no more.
% Education College or Above	The percentage of residents with at least a 4-year degree.
% Education Graduate	The percentage of residents with a graduate degree.
% Education Highschool	The percentage of residents with a high school diploma but no more.
% Education Less Highschool	The percentage of residents with less than a high school education.
% Education Some College	The percentage of residents with some college but no more.
% Family Dual Income	The percentage of families with dual income earners.
Average Family Size	The average size of resident families.
% Farmer	The percentage of households reporting farm income on their 2016 IRS tax return.
% Health Uninsured	The percentage of residents who report not having health insurance.
% Hispanic	The percentage of residents who report being Hispanic.
% Home Ownership	Percentage of households that own (rather than rent) their residence.
Median Home Value	The median value of homes that are owned by residents.
Housing Units	The number of housing units (or households) in the zip code.
% Income Household $150K Over	The percentage of households with income over $150,000.
Median Household Income	Median household income in USD.
% Household Income Under $5K	The percentage of households with income under $5,000.
Median Individual Income	The median income of individuals in the zip code.
% Labor Force Participation	The percentage of residents 16 and older in the labor force.
% Limited English	The percentage of residents who only speak limited English.
% Male	The percentage of residents who report being male.
% Married	The percentage of residents who report being married.
% Never Married	The percentage of residents never married.
Population	An estimate of the zip code's population.
% Race Asian	The percentage of residents who report their race as Asian.
% Race Black	The percentage of residents who report their race as Black or African American.
% Race Native	The percentage of residents who report their race as American Indian and Alaska Native.

% Race Other	The percentage of residents who report their race as Some other race.
% Race Pacific	The percentage of residents who report their race as Native Hawaiian and Other Pacific Islander.
% Race White	The percentage of residents who report their race White.
Median Rent Burden	The median rent as a percentage of the median renter's household income.
Median Rent	The median rent paid by renters.
% Self Employed	The percentage of households reporting self-employment income on their 2016 IRS tax return.
Unemployment Rate	The percentage of residents unemployed.
% Veteran	The percentage of residents who are veterans.

2. **Model Specifications:**

   2.1 Multiple provider episodes

   Model:

   glm(formula = overlap_prescriber ~ density + age_median + male +
   married + never_married + income_household_median + income_household_150_over +
   income_individual_median + home_ownership + housing_units +
   home_value + education_less_highschool + education_highschool +
   education_some_college + education_graduate + unemployment_rate +
   farmer + race_asian + race_native + race_pacific + race_other +
   hispanic + health_uninsured + charitable_givers, data = na.omit(df_merge))

   Coefficients:

Variable	Estimate	Std. Error	t Value	Pr(>|t|)	
(Intercept)	508.800	226.400	2.248	0.025	*
Density	-0.027	0.004	-7.150	0.000	***
Median Age	-5.562	1.521	-3.656	0.000	***
% Male	-7.593	1.949	-3.896	0.000	***
% Married	-8.721	1.803	-4.836	0.000	***
% Never Married	-8.163	2.033	-4.015	0.000	***
Median Household Income	0.002	0.001	2.101	0.036	*
% Income Household $150K Over	3.793	1.705	2.225	0.026	*
Median Individual Income	-0.002	0.001	-1.715	0.087	.
% Home Ownership	1.388	0.736	1.887	0.059	.
Housing Units	0.057	0.001	54.791	0.000	***
Median Home Value	0.000	0.000	-2.215	0.027	*
% Education Less Highschool	12.030	1.626	7.401	0.000	***
% Education Highschool	10.710	1.558	6.876	0.000	***
% Education Some College	10.750	1.652	6.509	0.000	***
% Education Graduate	3.264	1.847	1.768	0.077	.
Unemployment Rate	-3.817	1.920	-1.988	0.047	*
% Farmer	-11.210	3.537	-3.169	0.002	**
% Race Asian	-5.545	0.573	-9.678	0.000	***
% Race Native	-4.261	1.951	-2.184	0.029	*
% Race Pacific	54.950	11.770	4.669	0.000	***

% Race Other	-1.596	0.825	-1.935	0.053	.
% Hispanic	-2.847	0.680	-4.187	0.000	***
% Health Uninsured	-9.388	2.150	-4.366	0.000	***
% Charitable Givers	3.068	1.251	2.453	0.014	*

*Significance codes: 0 '***' 0.001 '**' 0.01 '*' 0.05 '.' 0.1 ' ' 1*

2.2 Overlapping opioid prescription for ≥ 7 days

Model:

glm(formula = IsoverlapOpioid ~ population + density + age_median +
married + never_married + income_household_150_over + income_individual_median +
home_ownership + housing_units + home_value + rent_burden +
education_less_highschool + education_bachelors + education_graduate +
labor_force_participation + farmer + race_white + race_asian +
race_pacific + hispanic + disabled + health_uninsured + charitable_givers,
data = na.omit(df_merge))

Coefficients:

Variable	Estimate	Std. Error	t Value	Pr(>|t|)	
(Intercept)	834.900	143.400	5.821	0.000	***
Population	-0.001	0.001	-1.761	0.078	.
Density	-0.013	0.003	-5.196	0.000	***
Median Age	-6.720	1.033	-6.505	0.000	***
% Married	-4.752	1.246	-3.813	0.000	***
% Never Married	-5.753	1.340	-4.292	0.000	***
% Income Household $150K Over	2.025	0.908	2.232	0.026	*
Median Individual Income	-0.001	0.001	-1.744	0.081	.
% Home Ownership	0.664	0.469	1.416	0.157	
Housing Units	0.036	0.003	13.662	0.000	***
Median Home Value	0.000	0.000	-1.657	0.098	.
Median Rent Burden	-0.826	0.532	-1.554	0.120	
% Education Less Highschool	-1.493	0.862	-1.732	0.083	.
% Education Bachelors	-6.076	0.970	-6.266	0.000	***
% Education Graduate	-4.213	0.864	-4.878	0.000	***
% Labor Force Participation	-1.223	0.778	-1.572	0.116	
% Farmer	-8.295	2.359	-3.516	0.000	***
% Race White	1.425	0.404	3.532	0.000	***
% Race Asian	-1.825	0.550	-3.316	0.001	***
% Race Pacific	15.630	7.929	1.971	0.049	*
% Hispanic	-0.886	0.436	-2.030	0.043	*
% Disabled	2.087	1.442	1.447	0.148	
% Health Uninsured	-4.329	1.429	-3.030	0.002	**
% Charitable Givers	4.039	0.858	4.709	0.000	***

*Significance codes: 0 '***' 0.001 '**' 0.01 '*' 0.05 '.' 0.1 ' ' 1*

2.3 Overlapping opioid and benzodiazepine for ≥ 7 days

Model:

glm(formula = IsoverlapOpioidBenzo ~ population + density + age_median + male + married + never_married + income_individual_median + housing_units + rent_burden + education_less_highschool + education_bachelors + education_graduate + labor_force_participation + self_employed + farmer + race_black + race_asian + race_native + race_other + disabled + commute_time + health_uninsured + veteran + charitable_givers, data = na.omit(df_merge))

Coefficients:

Variable	Estimate	Std. Error	t Value	Pr(>|t|)	
(Intercept)	1556.000	227.000	6.854	0.000	***
Population	-0.006	0.001	-4.918	0.000	***
Density	-0.019	0.004	-4.942	0.000	***
Median Age	-7.747	1.599	-4.845	0.000	***
% Male	-3.130	2.090	-1.498	0.134	
% Married	-5.396	1.981	-2.724	0.007	**
% Never Married	-6.671	2.231	-2.990	0.003	**
Median Individual Income	-0.003	0.001	-3.134	0.002	**
Housing Units	0.065	0.004	16.592	0.000	***
Median Rent Burden	-2.440	0.860	-2.839	0.005	**
% Education Less Highschool	-3.906	1.255	-3.111	0.002	**
% Education Bachelors	-9.884	1.525	-6.483	0.000	***
% Education Graduate	-8.296	1.310	-6.332	0.000	***
% Labor Force Participation	-2.642	1.231	-2.146	0.032	*
% Self Employed	4.537	1.729	2.623	0.009	**
% Farmer	-12.630	3.728	-3.388	0.001	***
% Race Black	-5.359	1.021	-5.247	0.000	***
% Race Asian	-4.385	0.590	-7.432	0.000	***
% Race Native	-4.888	2.046	-2.389	0.017	*
% Race Other	-2.923	0.794	-3.683	0.000	***
% Disabled	5.230	2.308	2.266	0.024	*
Median Commute Time	-2.229	1.053	-2.118	0.034	*
% Health Uninsured	-5.722	2.304	-2.484	0.013	*
% Veteran	-11.500	3.134	-3.669	0.000	***
% Charitable Givers	10.750	1.240	8.674	0.000	***

*Significance codes: 0 '***' 0.001 '**' 0.01 '*' 0.05 '.' 0.1 ' ' 1*

2.4 High standardized dosage of opioid prescriptions

Model:

glm(formula = IshighMME ~ population + age_median + married + never_married + income_household_150_over + income_individual_median + home_ownership + housing_units + education_highschool + education_bachelors + education_graduate + self_employed + farmer + race_asian + race_native + race_pacific + hispanic + disabled + limited_english + commute_time + health_uninsured, data = na.omit(df_merge))

Coefficients:

Variable	Estimate	Std. Error	t Value	Pr(>|t|)	
(Intercept)	286.700	60.020	4.777	0.000	***
Population	-0.001	0.000	-3.802	0.000	***
Median Age	-1.776	0.453	-3.916	0.000	***
% Married	-1.376	0.573	-2.403	0.016	*
% Never Married	-1.700	0.609	-2.791	0.005	**
% Income Household $150K Over	1.744	0.363	4.800	0.000	***
Median Individual Income	0.000	0.000	-1.539	0.124	
% Home Ownership	0.468	0.188	2.490	0.013	*
Housing Units	0.014	0.001	12.128	0.000	***
% Education Highschool	-0.997	0.460	-2.166	0.031	*
% Education Bachelors	-2.682	0.467	-5.746	0.000	***
% Education Graduate	-1.996	0.417	-4.786	0.000	***
% Self Employed	0.839	0.483	1.737	0.083	.
% Farmer	-2.730	1.069	-2.555	0.011	*
% Race Asian	-1.272	0.186	-6.826	0.000	***
% Race Native	-0.994	0.586	-1.696	0.090	.
% Race Pacific	7.184	3.574	2.010	0.045	*
% Hispanic	-0.632	0.175	-3.614	0.000	***
% Disabled	1.202	0.635	1.893	0.059	.
% Limited English	-0.592	0.314	-1.887	0.059	.
Median Commute Time	-0.422	0.294	-1.437	0.151	
% Health Uninsured	-2.329	0.658	-3.541	0.000	***

*Significance codes: 0 '***' 0.001 '**' 0.01 '*' 0.05 '.' 0.1 ' ' 1*

3. QQ-Plots

Figure S1: QQ-Plot of Overlapping Opioid Prescription for ≥ 7 Days:

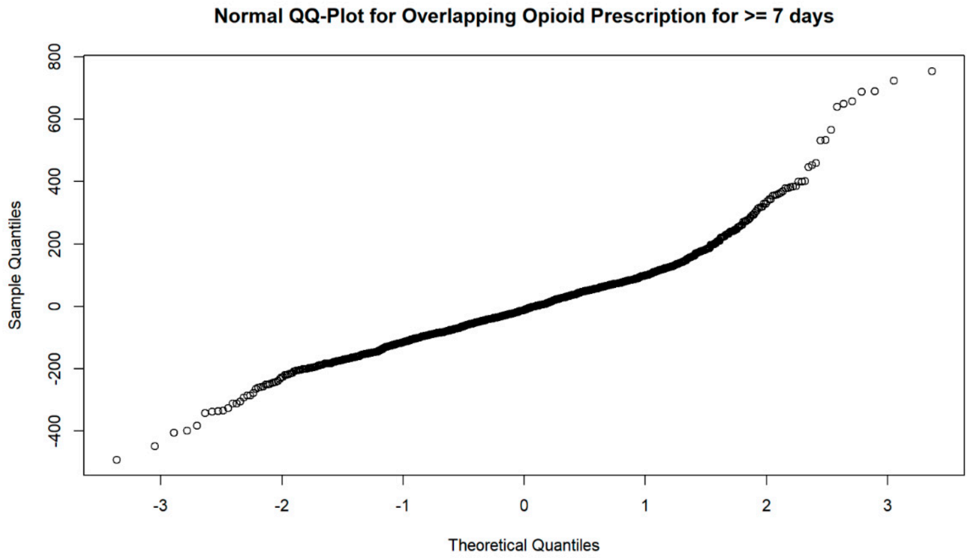

Figure S2: QQ-Plot of Overlapping Opioid and Benzodiazepine for ≥ 7 days:

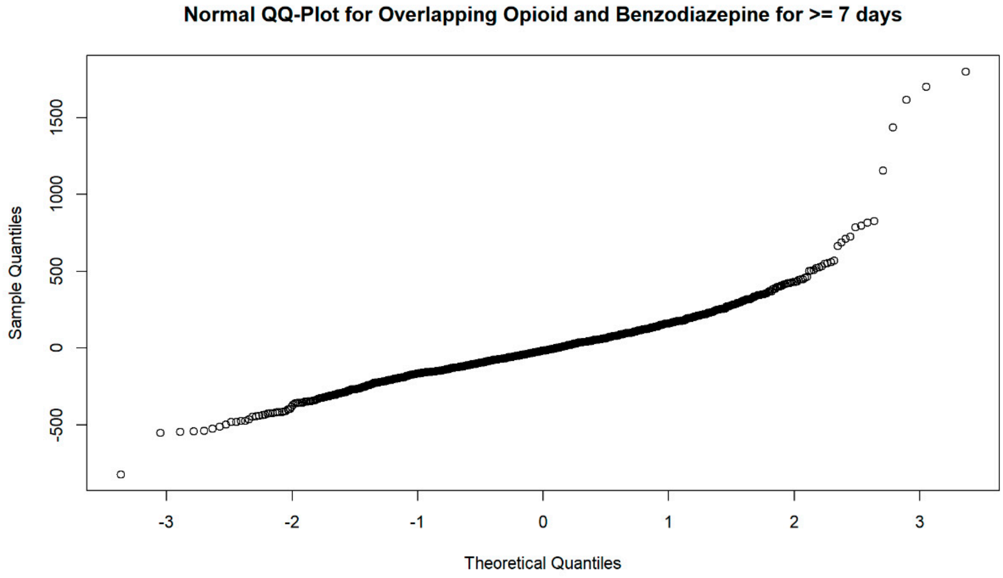

Figure S3: QQ-Plot of Multiple Provider Episodes:

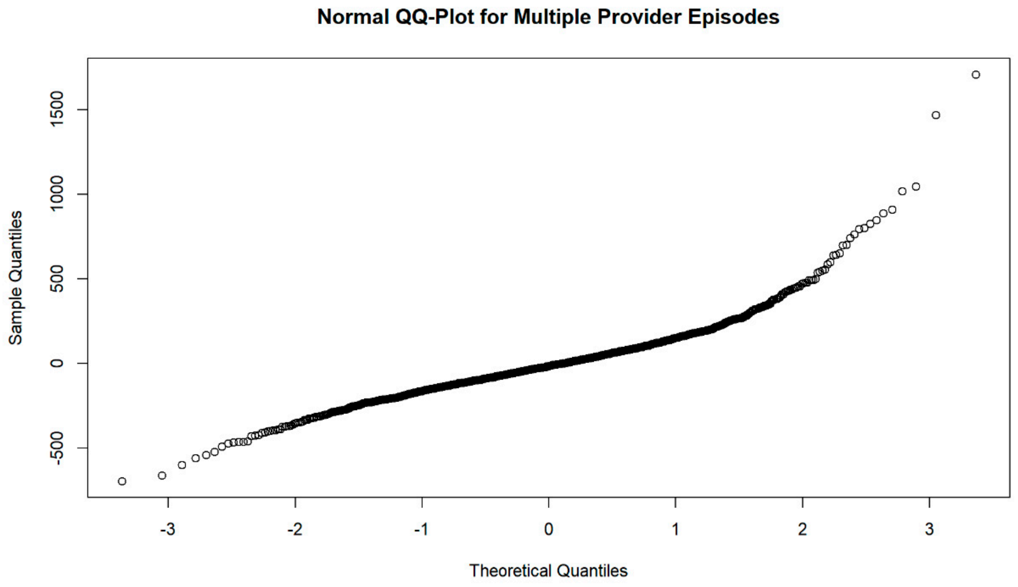

Figure S4: QQ-Plot of High standardized Dosage of Opioid Prescriptions:

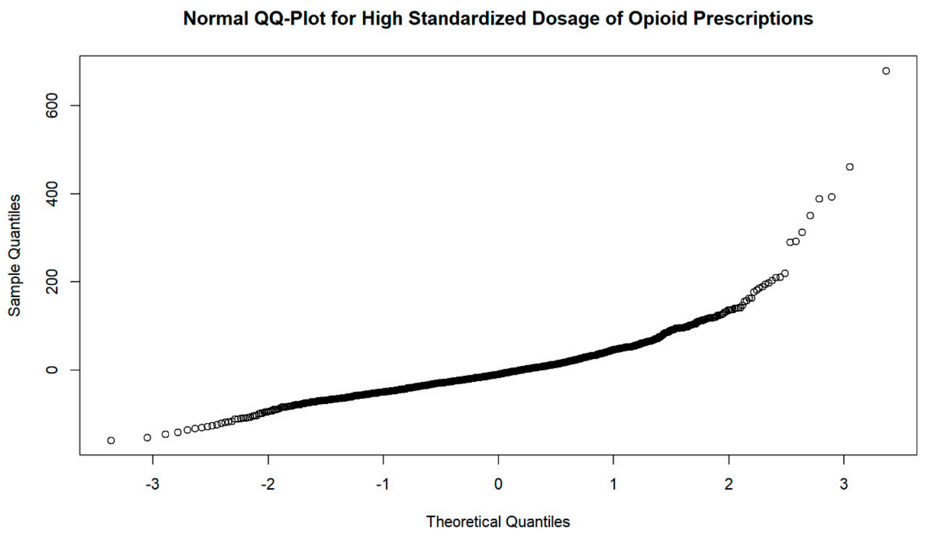

Supplement: Supplementary file 1 [file healthcare-11-01732-s001.zip › healthcare-2384924-supplementary.pdf]
